# Denosumab-Induced Severe Hypocalcaemia in Chronic Kidney Disease

**DOI:** 10.1155/2018/7384763

**Published:** 2018-11-04

**Authors:** Ryan Jalleh, Gopal Basu, Richard Le Leu, Shilpanjali Jesudason

**Affiliations:** ^1^Central and Northern Adelaide Renal and Transplantation Service, Royal Adelaide Hospital, Adelaide, South Australia, Australia; ^2^Department of Medicine, University of Adelaide, South Australia, Australia

## Abstract

**Background:**

Hypocalcaemia is increasingly recognized as a complication of denosumab use in Chronic Kidney Disease (CKD) patients with osteoporosis. Despite Therapeutic Goods Administration (TGA) notifications in 2013, we have subsequently encountered several cases of denosumab-induced hypocalcaemia, raising concern about lack of widespread awareness among prescribing practitioners.

**Aims:**

We reviewed the morbidity and healthcare intervention needs of CKD patients with hypocalcaemia attributed to denosumab.

**Methods:**

A retrospective case series of CKD patients with clinically significant hypocalcaemia after exposure to denosumab, encountered at the tertiary care referral hospital from December 2013 to February 2017, was undertaken.

**Results:**

Eight patients (52-85 years of age) with stage 4-5 CKD developed clinically significant hypocalcaemia (corrected calcium 1.45±0.21mmol/L) following denosumab therapy for osteoporosis. Seven of the eight patients required inpatient management with three patients requiring intravenous calcium replacement and cardiac monitoring in a high dependency unit. Our study also identified additional factors that could potentially contribute to hypocalcaemia such as lack of calcium supplementation, use of noncalcium based phosphate binders, absence of or use of lower doses of calcitriol supplementation, low vitamin D levels, concomitant treatment with loop diuretics, history of parathyroidectomy, or presence of acute medical illness.

**Conclusion:**

Multiple cases of severe hypocalcaemia in CKD patients following denosumab exposure were encountered after TGA warnings, resulting in considerable morbidity and intensive healthcare interventions in CKD patients. We advocate greater awareness amongst the medical profession, careful consideration before using denosumab in CKD patients, and close follow-up after administration to prevent morbidity.

## 1. Introduction

Denosumab is a fully humanised monoclonal antibody used for treatment of osteoporosis. Denosumab binds to the ligand to the receptor activator of nuclear factor kappa B (RANK-L)[1], the osteoclast differentiating factor, thereby preventing RANK-L from binding to RANK, consequently inhibiting downstream pathways of osteoclast formation, activity, and survival. Thus, it decreases bone resorption, increases bone mineral density (BMD), and reduces the risk of fragility fractures. It is a useful alternative to bisphosphonates in management of osteoporosis especially in patients who are intolerant or unresponsive to bisphosphonates. Unlike bisphosphonates, denosumab is not renally cleared, and hence its dosing is simplified in chronic kidney disease (CKD) patients in whom bisphosphonates are considered contraindicated [2]. In addition, its ease of administration as a subcutaneous injection has rapidly raised its popularity among clinicians.

However, the use of denosumab in CKD patients is not without risk. Hypocalcaemia is a known adverse event with incidence 14-15% reported among CKD patients [3, 4]. The Therapeutic Goods Administration (TGA) has issued medication safety updates of hypocalcaemia in 2013 and additionally of prolonged QT interval due to hypocalcaemia in 2016 [5, 6]. However, there is limited information for the safety in patients with CKD and consequently several cases of patients with hypocalcaemia after denosumab were encountered in the recent years [3, 7]. This case series was conducted to highlight the morbidity and healthcare interventions needed to manage this complication and thereby raise awareness of the risk of hypocalcaemia in CKD patients receiving denosumab.

## 2. Materials and Methods

We performed a retrospective case series of patients with CKD stage 4 or 5, who developed clinically significant hypocalcaemia following denosumab therapy and presented to a tertiary hospital between December 2013 and February 2017. Cases were referred by nephrologists at the institution who had patients with CKD stage 4 or 5, had at least one dose of subcutaneous denosumab 60mg, and had hypocalcaemia (corrected calcium <2.10) identified at any time following the dose of denosumab. The clinical data, collected by review of medical records and in liaison with the primary care general practitioners, included stage of chronic kidney disease, profile of corrected calcium, phosphate levels, parathyroid hormone levels, alkaline phosphatase (ALP), vitamin D levels, T-score from the most recent dual-energy X-ray absorptiometry (DEXA) scan preceding the denosumab, dosing history, and time of onset of hypocalcaemia. This study was approved by our institution's Human Research Ethics Committee.

The calcium level was corrected for low serum albumin using the following formula [9]:

(1) Corrected  calcium mmol/L=total  serum  calcium mmol/L+0.02 40−serum  albumin g/L

## 3. Results

A total of eight CKD patients with symptomatic hypocalcaemia were identified from Dec 2013 to Feb 2017 aged between 52 and 85 years. A summary of the biochemical parameters and the patient's current calcium/vitamin D therapy are described in Tables 1 and 2.

The number of doses of denosumab before developing hypocalcaemia varied from a single dose in most patients up to 6 doses. The onset of hypocalcaemia from the last dose of denosumab ranged from 2 to 12 weeks with a mean of 6.6 weeks (median 8 weeks).

**Figure 1:**
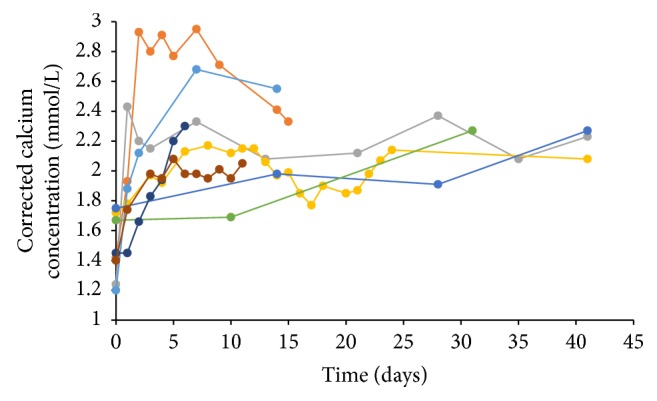
Corrected calcium levels on admission day (time 0) and subsequently (n=8 cases).

Seven of the eight patients required hospital admission. Calcium levels at and after admission are shown in Figure 1. Three patients required intravenous calcium replacement with cardiac monitoring in an intensive care/high dependency unit. Four patients were managed in the ward and one patient was managed as an outpatient.

Four of the eight patients were not receiving regular calcium supplementation, which is associated with an increased risk of severe hypocalcaemia [7]. All three cases that required high dependency support were not on concurrent calcium supplements when commenced on denosumab. Two of the three patients admitted to a high dependency unit were on regular furosemide for management of congestive cardiac failure. The increased calcium loss via diuresis may have increased their risk of hypocalcaemia. Five out of eight cases were on a less than daily dosing of calcitriol and four out of eight cases had low levels of vitamin D. None of the patients were receiving cinacalcet.

Seven out of 8 patients had additional factors that could potentially contribute or worsen denosumab-related hypocalcaemia including use of noncalcium based phosphate binders (1 case), absence of or use of low doses of calcitriol (less than daily doses) supplementation (5 cases), low levels of vitamin D (4 cases), concomitant treatment with loop diuretics (2 cases), history of parathyroidectomy (1 case), and presence of an acute medical illness with reduced oral intake (1 case).

There was no significant correlation between time from denosumab dose to hypocalcaemia and the severity of hypocalcaemia (Pearson correlation coefficient r = 0.19, r^2^ = 0.036 (p = 0.65)). There was no correlation between calcium level at presentation and length of stay. All patients required close outpatient monitoring of calcium levels on discharge with frequent medical follow-up encounters. In all cases, following the presentation of hypocalcaemia, the use of denosumab was discouraged.

## 4. Discussion

Chronic kidney disease, mineral and bone disorder (CKD-MBD), is frequently present in stage 4 to 5 CKD and can cause fragility fractures as well as a low bone mineral density.[10] In CKD stage 4-5 patients, a history of fragility fractures and DEXA results is unable to differentiate osteoporosis from CKD-MBD. However, the treatment of CKD-MBD is different from that of osteoporosis. This distinction is difficult but important as denosumab and other antiresorptive agents may not be beneficial in the setting of CKD-MBD. The gold standard of discriminating between the various forms of renal bone disease is transiliac bone biopsy performed with prior double tetracycline labelling.[10] However, due to cost and invasiveness, this procedure is not frequently done in clinical practice and none of the cases reported above underwent this form of testing.

**Figure 2:**
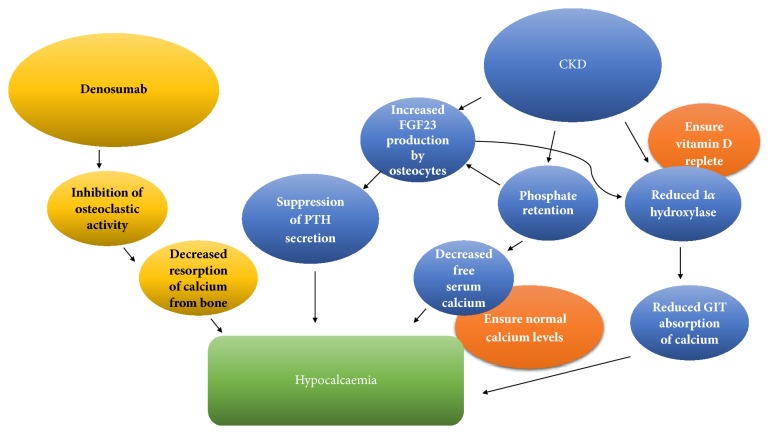
A summary of mechanisms for increased risk of hypocalcaemia with denosumab in patients with CKD.

As CKD progresses, there is decreased phosphate clearance and this is thought to be the driving mechanism that results in increased production of fibroblast growth factor-23 (FGF-23) mainly by osteocytes.[11–13] In addition to reduced production of 1*α* hydroxylase in CKD due to decreased functional renal tissue, there is also inhibition of the enzyme due to FGF-23 accumulation.[11] Ultimately, this results in reduced absorption of calcium from the gastrointestinal tract potentially exacerbating hypocalcaemia. In addition, FGF-23 suppresses PTH secretion which may impair the ability of the kidneys to maintain calcium homeostasis [14]. There are multiple mechanisms that predispose CKD patients to hypocalcaemia (Figure 2).

Denosumab downregulates osteoclast activity in CKD patients, increasing their predisposition to hypocalcaemia. Many patients with CKD could already be suffering from low bone turnover and denosumab could potentially worsen this and possibly increase the risk of fractures. There is a paucity of evidence for the use of denosumab in CKD stage 4 to 5 and it is currently unclear if denosumab provides benefit. Significantly, in the original denosumab trial, the FREEDOM study [1, 15], only 73 of 7808 patients recruited had CKD stage 4 and none of the patients had CKD stage 5. Patients with secondary hyperparathyroidism, associated with the CKD stage 4 to 5 population, were also excluded from the study. Thus, the results from FREEDOM study are clearly not widely applicable for patients with advanced CKD and those with secondary hyperparathyroidism.

Despite the lack of robust evidence of benefit, and warnings of possible harm, denosumab continues to be used in patients with advanced CKD and cases of hypocalcaemia secondary to denosumab use continue to be reported, as demonstrated by this case series. The cases described outline the potentially life-threatening severity of morbidity that may occur. Most of our patients required hospitalisation, with half requiring intensive care unit admissions for intravenous calcium replacement and cardiac monitoring. Importantly, significant hypocalcaemia often occurred some weeks after denosumab administration. These cases highlight the unpredictability of hypocalcaemia and the need for heightened awareness and closer biochemical monitoring for CKD patients receiving denosumab. A critical review of the patients' comorbidities and other medications that regulate calcium and parathyroid hormone to identify risk factors for hypocalcaemia (or impaired response to hypocalcaemia) is important before considering denosumab use in patients with CKD.

The denosumab product information [16] includes a special warning regarding hypocalcaemia and states that this must be corrected prior to initiating therapy. Consistent with the findings in this case series, it warns that patients with the following may have a predisposition to hypocalcaemia: severe renal impairment, history of hypoparathyroidism, thyroid or parathyroid surgery, malabsorption syndromes, and excision of small intestine. It recommends monitoring of calcium in these cases especially within the first two weeks of initiating therapy and prior to each dose. Similar to our recommendations, it also advises that all patients should be instructed on the symptoms of hypocalcaemia and the importance of maintaining calcium levels with adequate calcium and vitamin D supplementation. In addition to following the recommendations on the product information, based on our experience, we have developed further clinical practice points for clinicians considering denosumab therapy in patients with advanced CKD, which may prevent serious hypocalcaemia. (Box 1). This approach is supported by a pilot study by Chen et al. [17]. In this study, 12 patients on renal replacement therapy received denosumab for treatment of secondary hyperparathyroidism. Consistent with our findings, = a high risk of hypocalcaemia in patients with CKD is noted; however with aggressive calcium and calcitriol supplementation, use of high calcium dialysate, and weekly blood tests monitoring calcium in the first month of initiating denosumab, none of the patients needed inpatient admission for management of hypocalcaemia.

A limitation of this study is that it is a retrospective, observational study with a small number of reported patients. The total incidence of hypocalcaemia among all CKD patients receiving denosumab was not known and there was a lack of information for why denosumab was administered to the patients. Furthermore, patients with asymptomatic hypocalcaemia where biochemical monitoring was not undertaken could not be identified. Further research is required to determine the optimal regime for early identification and prevention of hypocalcaemia in patients with chronic kidney disease receiving denosumab.

## 5. Conclusions

Denosumab can cause hypocalcaemia in patients with CKD. Given the number of cases that we have observed, there may be a lack of awareness of the risk of hypocalcaemia, the need for close monitoring of calcium levels, and the paucity of evidence of the effectiveness of denosumab in CKD stages 4-5. We urge clinicians to exercise caution when using denosumab in patients with advanced CKD, to consult with the patient's nephrologist when initiating treatment, and to arrange biochemical monitoring after administration.

## Figures and Tables

**Box 1:**
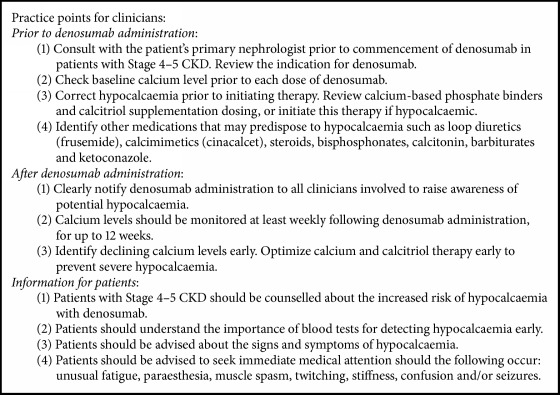
Based on the authors' experience, our recommended practice points while considering denosumab in CKD patients to address potential hypocalcaemia.

**Table 1:** Baseline clinical data for 8 patients with CKD stages 4 to 5 who were on denosumab treatment.

**Case**	**Sex**	**CKD stage**	**Serum Creatinine** **(**µ**mol/L)**	**eGFR** **(ml/min/1.73m** ^**2**^ **)**	**Ca2+ prior to denosumab**	**Corr Ca** ^**2+**^ **(Ref: 2.10 – 2.55 mmol/L) at presentation**	**P** **O**_4_^3−^ **(Ref: 0.65 – 1.45 **mmol**/L)**	**PTH** **(Ref: 0.8 – 5.5 pmol/L)**	**ALP** **(Ref: 30 – 110 units/L)**	**Vitamin D** **(Ref: 60 – 160 nmol/L)**	**T-score**
**1**	F	5	394	11	2.21	1.42	1.85	55.4	61	75	-3.0
**2**	F	4	207	20	2.28	1.24	1.02	83.9	77	27	-
**3**	M	4	289	18	2.11	1.30	2.63	58.2	399	45	-2.5
**4**	F	5	645	5	2.20	1.20	2.71	80.1	54	54	-2.5
**5**	F	5D	601	5	2.44	1.80	1.68	59.6	74	90	-3.0
**6**	F	4T	221	20	2.18	1.70	1.65	15.5	93	62	-1.3†
**7**	F	4T	234	18	2.38	1.50	0.69	-	101	88	-4.4
**8**	M	5	410	11	2.14	1.40	1.40	33.3	76	32	-2.7

5D = stage 5 CKD on dialysis and 4T = stage 4 CKD with a renal transplant. All results (including serum phosphate, PTH, ALP, and vitamin D) were obtained closest to the time of presentation with hypocalcaemia in patients who were symptomatic or nadir of hypocalcaemia in patients who were asymptomatic and being actively monitored. All T-scores were obtained based on bone densitometry of the femur except for case 6† who had forearm bone densitometry due to bilateral hip joint replacements. eGFR was calculated using the CKD-EPI formula [8]; Ref = reference range.

**Table 2:** Baseline clinical data for 8 patients with CKD stages 4 to 5 who were on denosumab treatment.

**Case**	**Number of denosumab doses**	**Length of hospital stay**	**Ward/HDU/Outpatient**	**Time from dose of denosumab to calcium nadir (weeks)**	**Time to normal calcium level (weeks)**	**Calcium dose regime prior to admission**	**Calcitriol dose regime prior to admission**	**History of fragility fracture**
1	1	2	Ward	12	2	1g TDS	250ng twice weekly	No
2	1	4	HDU	4	8	Nil	Nil	Yes
3	1	25	Ward	8	11	1g TDS	250ng daily	No
4	3	11	HDU	8	7	Nil	250ng alternate daily	Yes
5	4	1	Outpatient	9	8	500mg TDS	250ng thrice weekly	No
6	6	7	Ward	8	2	Nil	250ng daily	No
7	1	8	Ward	2	1	500mg TDS	250ng daily	No
8	1	10	HDU	2	1	Nil	Nil	No
